# Adult Human Brain Tissue Cultures to Study NeuroHIV

**DOI:** 10.3390/cells13131127

**Published:** 2024-06-29

**Authors:** Rachel Van Duyne, Elena Irollo, Angel Lin, James A. Johnson, Alain M. Guillem, Erick V. O’Brien, Laura Merja, Bradley Nash, Joshua G. Jackson, Atom Sarkar, Zachary A. Klase, Olimpia Meucci

**Affiliations:** 1 Department of Pharmacology and Physiology, Drexel University College of Medicine, Philadelphia, PA 19102, USA; 2 Department of Neurosurgery, Drexel University College of Medicine, Philadelphia, PA 19102, USA; 3 Global Neurosciences Institute, LLC, Philadelphia, PA 19107, USA; 4 Center for Neuroimmunology and CNS Therapeutics, Institute for Molecular Medicine and Infectious Diseases, Drexel University College of Medicine, Philadelphia, PA 19102, USA; 5 Department of Microbiology and Immunology, Drexel University College of Medicine, Philadelphia, PA 19102, USA

**Keywords:** HIV-associated neurocognitive disorders (HAND), human brain organotypic slice cultures, HIV-1 infection, dendritic spine analysis, microelectrode array

## Abstract

HIV-associated neurocognitive disorders (HAND) persist under antiretroviral therapy as a complex pathology that has been difficult to study in cellular and animal models. Therefore, we generated an ex vivo human brain slice model of HIV-1 infection from surgically resected adult brain tissue. Brain slice cultures processed for flow cytometry showed >90% viability of dissociated cells within the first three weeks in vitro, with parallel detection of astrocyte, myeloid, and neuronal populations. Neurons within brain slices showed stable dendritic spine density and mature spine morphologies in the first weeks in culture, and they generated detectable activity in multi-electrode arrays. We infected cultured brain slices using patient-matched CD4+ T-cells or monocyte-derived macrophages (MDMs) that were exposed to a GFP-expressing R5-tropic HIV-1 in vitro. Infected slice cultures expressed viral RNA and developed a spreading infection up to 9 days post-infection, which were significantly decreased by antiretrovirals. We also detected infected myeloid cells and astrocytes within slices and observed minimal effect on cellular viability over time. Overall, this human-centered model offers a promising resource to study the cellular mechanisms contributing to HAND (including antiretroviral toxicity, substance use, and aging), infection of resident brain cells, and new neuroprotective therapeutics.

## 1. Introduction

Human immunodeficiency virus type 1 (HIV-1) continues to pose significant challenges worldwide, with an estimated 39.0 million people living with the virus in 2022 [1]. While HIV-1 treatment has been revolutionized by antiretroviral therapy, many people with HIV still present with a range of cognitive and neurologic symptoms termed HIV-associated neurocognitive disorders (HAND) [2]. HAND symptoms are generally less severe in people using antiretroviral therapy, but treatment does not fully prevent the more subtle forms of HAND that still disrupt quality of life [3]. Thus, HAND remains an important and unmet medical need that is increasing in prevalence as people with HIV live longer with the virus [4,5].

Previous work has highlighted factors that likely contribute to HAND symptoms, including but not limited to chronic low-level inflammation [6,7], continued expression of neurotoxic viral proteins [8,9], defective proviruses [10,11], and possibly long-term exposure to select ART regimens [12,13,14]. Furthermore, HAND symptoms can be aggravated by substance use [15], particularly opioids [16,17,18] and stimulants [19,20], which is more common in people with HIV [21,22]. This creates a complicated picture where HAND may be driven by many factors that interact in nuanced ways in the human brain. Much of our current knowledge of HAND comes from a combination of clinical studies of people with HIV and laboratory studies that use cellular and animal model systems of HIV-1 [23]. Clinical studies provide valuable insights into the structural and functional changes in the brain associated with HIV-1 infection but lack the ability to study disease mechanisms outside clinical trials. On the contrary, cellular and animal model systems of HIV-1 allow robustly controlled studies of the underlying disease mechanisms associated with HIV-1 infection of the brain, but they do not capture the full complexity of the human brain [24]. This disconnect could be part of the reason we currently lack an effective adjuvant therapy to treat HAND [25,26]. 

We aimed to address this disconnect by developing an adult human brain slice culture model of HIV-1 infection, which would enable controlled studies of disease mechanisms in a more physiologically relevant context. The model was developed using donated neurosurgical tissue resections and donor-matched peripheral blood from two hospitals in the Greater Philadelphia region. Importantly, our brain slice culture system only used healthy brain tissues from the supratentorial neocortical parenchyma that were removed to access deeper lesions that required surgery. The slice cultures preserved the brain’s architecture and maintained high cellular viability for several weeks, allowing for studies of HIV-1 infection over time in the same brain slice. Our model used a unique HIV-1 infection strategy where slice cultures were infected using the same tissue donor’s peripheral immune cells, thereby reflecting the trojan horse strategy [27,28] by which HIV-1 can enter the brain. We collected whole blood at the time of surgery and isolated T-cells and macrophages, infected these cells with a fluorescent HIV-1 in vitro, and seeded these cells directly on the donor-matched slice cultures. This strategy successfully infected cultures with HIV-1 and allowed us to measure viral replication and cellular-level infection via different techniques. Furthermore, antiretroviral therapy strongly suppressed infection in the brain slice cultures, suggesting this system can model HIV-1 brain infection in the modern era. Although still at an early stage of development, this model offers an impactful tool to study the mechanisms of neuronal injury in the HIV-1-infected brain, examine the contributions of antiretrovirals and/or HIV-1 comorbidities, monitor infection of resident cells, and test potential neuroprotective agents.

## 2. Materials and Methods

### 2.1. Patient Recruitment

All patient participation was voluntary and obtained only after extensive discussion and then signing IRB-sanctioned written consent forms. Chain of Custody Tissue Tracking documentation traveled with the sample from the operating room to the laboratory. This study was conducted in accordance with the Declaration of Helsinki, and the protocol was approved by the IRB committees from both participating hospitals: Crozer-Keystone Health System (IRB number #00006457) and Trinity Health Mid-Atlantic (IRB number #00012775 [active]). Patients that were positive for HIV, SARS-CoV-2, or other pathogens were not considered as potential donors in this study. All tissue samples are accompanied by de-identified demographic information and other relevant medical history. 

### 2.2. Collection of Brain Tissue, Blood, and Cerebrospinal Fluid

Human brain tissue and blood samples were collected during normally scheduled brain surgeries at two participating hospitals. Brain tissues selected for our study consisted exclusively of supratentorial neocortical parenchyma and did not use cerebellar tissue. Patients with cortical lesions were excluded. Our studies used cortical brain tissues from the incision path rather than around the tumor to better reflect healthy tissues. Furthermore, the vascularity of all tissue samples was meticulously preserved until the very moment they were removed, and resected brain tissues were submerged in ice-cold artificial cerebrospinal fluid (aCSF) within 10 s of resection for transport to the laboratory. Blood samples from each patient were collected into EDTA tubes (BD vacutainer 366643) and stored at 25 °C until processing. Both sets of tissues were transported to Drexel University College of Medicine within 30–60 min from the collection time. Human cerebrospinal fluid (hCSF) used in this study was sourced from one patient (a case of benign hydrocephalus not recruited for tissue donation), who donated hCSF over two days (processed separately, i.e., 24 h/each). This hCSF was constantly kept on ice during collection and was then centrifuged at 4000 rpm for 10 min at 4 °C and sterile filtered using a 0.2 μm pore size filter. Sterile hCSF was stored in aliquots of 15–50 mL at −80 °C until use in the slice culture medium.

### 2.3. Human Brain Slice Culture

All tools used to prepare slice cultures were sterilized using either an autoclave or UV light. All solutions/reagents were prepared before tissues arrived for processing. aCSF cutting solution (280 mM sucrose, 5 mM KCl, 2 mM MgCl_2_ · 6H_2_O, 1 mM CaCl_2_, 20 mM glucose, 10 mM HEPES, 5 mM Na-pyruvate, 3 mM thiourea, 2 mM Na-ascorbate) was prepared and stored in aliquots of 50 mL at −20 °C for no longer than 1–2 months. Human slice culture media (hSCM) (neurobasal medium, 2% B27 supplement, 1% N2 supplement, 1% L-Glutamine (×100), 0.5% glucose, 1% antibiotic/antimycotic (×100)) was prepared fresh the day before surgery and then on a weekly basis.

When brain tissues arrived at the laboratory, we removed capillaries, blood clots, debris, and any visibly damaged tissue. The remaining tissue was divided into several parts (depending on tissue size) while submerged in ice-cold cutting solution. Each part was then sliced into 200 μm-thick sections using a tissue chopper (McIlwain tissue chopper 800 series MCT/2), maximizing the amount of gray matter in each slice. Slices were then placed in a dish containing ice-cold aCSF, gently swirled, and carefully separated using needles. Slices with clear margins and no nicks were transferred to another dish filled with ice-cold aCSF and then rinsed in hSCM. Slices were then transferred into culture membrane inserts (0.4 μM, PTFE, hydrophilic membrane inserts Millipore-Sigma, Burlington, MA, USA, #PICM03050), placed in a 6-well plate, and kept at 37 °C and 5% CO_2_. This produces a semi-dry culture that maintains slices at the air/media interface with the hSCM in the bottom of the well. Up to five slices were placed in each insert, ensuring that slices did not overlap. The slice cultures were maintained in hSCM for the first two days. After 48 h, the hSCM was replaced with a 1:1 mixture of hSCM and hCSF. The media was changed every other day, and the slices were maintained in culture up to four weeks.

### 2.4. Tissue Clearing and Staining

Organotypic human brain slice cultures were cleared using a modification of the EZClear protocol [29]. Slices were fixed in situ with 4% paraformaldehyde for 30 min above and below the membrane support. After fixation, slices were rinsed 3× for 1 h in phosphate-buffered saline (PBS) with gentle shaking at 25 °C. Slices were then removed from the membrane supports with a scalpel and transferred to a glass liquid scintillation vial. Tissue lipids were removed by incubation with 50% (*v*/*v*) tetrahydrofuran (THF) (with 250 ppm butylated hydroxytoluene (BHT), Millipore-Sigma, Burlington, MA, USA, #186562) prepared in sterile Milli-Q H_2_O for 16 h. Slices were then rinsed with sterile PBS 4× for 1 h each at 25 °C to remove any trace of THF. Next, slices were moved to individual wells of a 24 well tissue culture dish for immunostaining as free-floating slices. Slices were stained with either chicken anti-GFAP (Abcam, Cambridge, UK, Cat#ab4674, RRID:AB_304558, 1:500), rabbit anti-MAP2 (Millipore, Burlington, MA, USA, Cat#ab5622, RRID:AB_91939, 1:500), mouse anti-TMEM 119 (R&D Systems, Minneapolis, MN, USA, Cat#FAB10313R-100UG, 1:100), or mouse anti-HIV-1 p24 gag (BEI Resources, Manassas, VA, USA, Cat#ARP-6521, 1:250) in a PBS solution with 0.4% Triton X-100 and 5% goat serum. Slices were then stained with appropriate secondary antibodies, including goat anti-chicken Alexa Fluor 488 (Thermo Fisher Scientific, Waltham, MA, USA, Cat# A-11039, RRID:AB_2534096, 1:400), goat anti-rabbit Alexa Fluor 546 (Thermo Fisher Scientific Cat# A-11035, RRID:AB_2534093, 1:400), or goat anti-mouse Alexa Fluor 568 (Thermo Fisher Scientific Cat#A-11004, RRID:AB_2534072, 1:400). Slices were incubated for up to 1 week in either secondary or primary antibodies. After antibody staining, slices were counterstained overnight with Hoechst 33342 solution (BD Pharmingen, Franklin Lakes, NJ, USA, Cat#561908, 10 µg/mL) and transferred to glass slides. We formed wells around each slice using a hydrophobic spacer (SecureSeal, ThermoFisher, Waltham, MA, USA, Cat# S24737) to ensure slices were not deformed during mounting. Each well received a Refractive Index matching solution (RI; EZView (80% Nycodenz (Accurate Chemical & Scientific, Carle Place, NY, USA, 100334-594), 7 M urea, 0.05% sodium azide prepared in 0.02 M sodium phosphate buffer)) and the slices equilibrated for 24 h prior to imaging.

### 2.5. Cell Culture

HEK293T cells (ATCC #CRL-3216) were cultured in Dulbecco’s Modified Eagle Medium (DMEM) (Corning, Corning, NY, USA, #15-013-CV) supplemented with 10% (*vol*/*vol*) heat-inactivated fetal bovine serum (Gibco, Waltham, MA, USA, #16140071) and 1× penicillin/streptomycin/L-glutamine (PSG) (ThermoFisher Scientific #10-378-016) at 37 °C and 5% CO_2_. Purified CD4+ T-cells were cultured in RPMI-1640 (Roswell Park Memorial Institute, Corning, Waltham, MA, USA, #15040CV) supplemented with 20% (*vol*/*vol*) fetal bovine serum, 1× PSG, and 5 U/mL human rIL-2 (NIH AIDS Reagent Program), RPMI-20/IL-2. CD4+ T-cell isolates were stimulated with 5 μg/mL phytohemagglutinin-P (PHA-P) (Sigma, Burlington, MA, USA, #L1668) for 48–72 h at 37 °C and 5% CO_2_ and subsequently cultured in RPMI-20/IL-2. Monocyte-derived macrophages (MDMs) were differentiated and cultured in a macrophage media consisting of DMEM supplemented with 10% (*vol*/*vol*) heat-inactivated fetal bovine serum, 1× PSG, 5% (*vol*/*vol*) human AB serum (Gemini Bio-Products, Sacramento, CA, USA, #100-512), 10 mM HEPES (N-2-hydroxyethylpiperazine-N′-2-ethanesulphonic acid) (Corning #25-060-Cl), and 10 ng/mL macrophage-colony stimulating factor (M-CSF) (PeproTech, Cranbury, NJ, USA, #300-25) at 37 °C and 5% CO_2_. MDMs were allowed to attach and differentiate by plastic adherence for 48–72 h, then unbound cells were washed away with macrophage wash media consisting of DMEM with 10 mM HEPES.

### 2.6. PBMC Isolation, CD4+ T-Cell Purification, and MDM Differentiation

Peripheral blood mononuclear cells (PBMCs) were isolated from whole blood samples using Ficoll (Fisher Scientific #45-001-751) density centrifugation (1.078 g/mL). Briefly, whole blood was spun at 1500 rpm for 5 min at 25 °C. Plasma was collected and stored in 1 mL aliquots at −80 °C. Plasma volume was replaced with PBS without Ca^2+^/Mg^2+^ and diluted blood (15 mL) was overlaid onto (13 mL) Ficoll. Samples were spun at 300× *g* for 30 min at 25 °C with no braking. PBMCs were collected from the interphase and washed with 2× volumes of PBS without Ca^2+^/Mg^2+^. PBMCs were spun at 1200 rpm for 5 min at 4 °C and counted. 1 × 10^6^ PBMCs were reserved for characterization by flow cytometry. The remainder of the PBMCs were used to isolate CD4+ T-cells.

CD4+ T-cells were purified from PBMCs using MACS positive isolation beads (Miltenyi, Bergisch Gladbach, Germany, #130-045-101) according to manufacturer’s recommendations. We also collected the negatively selected flow-through (CD4-depleted PBMCs), which contained monocytes. Less than 1 × 10^6^ cells were reserved from each population to determine isolate purity by flow cytometry. CD4+ T-cell isolates were cultured for 48–72 h at 1 × 10^6^ cells/well in RPMI-20/IL-2 media + PHA-P as indicated above.

CD4-depleted PBMCs were assumed to contain approximately 15% monocytes and were plated in macrophage media such that 1 × 10^6^ monocytes were plated per well of a 6-well plate and allowed to attach via plastic adherence. At 48–72 h post-plating, CD4+ T-cells were washed, counted, and replated in RPMI-20/IL-2 at approximately 1 × 10^6^ cells/well. At the same time, unbound CD4-depleted PBMCs were removed, attached MDMs were washed with macrophage wash media, and fresh macrophage media was added. Cells were then either uninfected or infected with HIV-1 as indicated.

### 2.7. Mechanical and Enzymatic Dissociation of Tissue Slices

At the indicated time points in culture or post-infection, tissue slices were collected from the culture insert using a wet paintbrush and were processed using the Adult Brain Dissociation Kit, mouse and rat (Miltenyi #130-107-677) on the gentleMACS Octo Dissociator with Heaters (Miltenyi #130-096-427) according to manufacturer’s recommendations. Volumes were scaled down to accommodate small tissue. Though the above dissociation kit is intended for mouse/rat brain, we have used it successfully for human and mouse with no discernable differences. Briefly, tissues were incubated with two enzymes and were mechanically dissociated using the gentleMACS program “37C_ABDK_02” for 30 min. Cells were then washed in PBS with Ca^2+^/Mg^2+^, filtered using a 70 μm filter, and pelleted. Cells were subjected to density gradient centrifugation to remove debris and myelin contamination. Lastly, residual red blood cells were lysed, cells were washed, and samples were either fixed in 1% formaldehyde or stained for flow cytometry.

### 2.8. Dendritic Spine Staining and Analysis

Slice cultures used for dendritic spine analysis were sectioned at 200 μm thickness and cultured for up to 4 weeks. Select cultures were fixed at days in vitro (DIV) 0, 7, 14, 21, and 28 with 4% paraformaldehyde for 10 min at 25 °C, then rinsed 3× in PBS and stored at 4 °C until beginning the staining protocol. We labeled neurons and dendritic spines using DiI stain, which was prepared as previously described [30]. To begin, 300 mg of tungsten beads (Bio-Rad, Hercules, CA, USA, #1652269) were suspended in 99.5% pure methylene chloride (Fisher Scientific D37) and sonicated in a water bath for 1 h. Then, we created the DiI solution by dissolving crystalized DiI (14.5 mg, Invitrogen, Waltham, MA, USA, #D282) in methylene chloride under protection from light. Tungsten beads were coated with DiI by first placing 100 μL of the bead solution on a glass slide and adding 100 μL of DiI solution on top with slow mixing via micropipette. The dried bead/dye mixture was scraped onto weighing paper with a razor blade, placed into a 15 mL conical tube with 3 mL distilled and deionized water, and sonicated in a water bath for 20 min. Then, the mixture was drawn into Tezfel tubing coated with polyvinylpyrrolidone (Fisher Scientific BP431-100) and dried with nitrogen gas for 1 h. Dry tubing was cut into 13 mm cartridges and loaded into the Helios Gene Gun (Bio-Rad), which delivered DiI-coated beads to slices through a 3 μm pore filter paper using pressurized helium gas (120 PSI). After delivery, slices were quickly washed 3× with PBS and stored overnight at 4 °C to allow DiI to diffuse through the tissue. Stained slices were mounted the next day using ProLong Gold Antifade Mountant (Invitrogen P36930) and stored in the dark at 4 °C until imaging.

Dendrites from human brain slices were imaged with an Olympus FLUOVIEW FV3000 confocal microscope using a 100× objective at 0.3 μm per Z-step. We imaged 3–4 human brain slices at each experimental timepoint and analyzed at least 3 dendrites per slice to average as single data point. Analyzed dendrites were at least 100 µm long and included both basal and apical dendrites. Dendrite micrographs were analyzed using Neurolucida 360 software (v.2021.1.1), which quantified dendritic spines and classified them into predefined morphologies (thin, mushroom, stubby, filopodia) [31].

### 2.9. Multielectrode Array (MEA) Electrophysiology

All solutions were oxygenated with carbogen (95% oxygen and 5% carbon dioxide) for at least 30 min before use with brain tissue. Immediately upon arrival to the laboratory, a small piece of brain tissue was removed for acute MEA recording. This tissue was washed in an oxygenated sucrose cutting solution composed of 150 mM sucrose, 40 mM NaCl, 4 mM KCl, 1.25 mM NaH_2_PO_4_·H_2_O, 0.5 mM CaCl_2_·2H_2_O, 7 mM MgCl_2_ · 6H_2_O, 10 mM glucose, and 26 mM NaHCO_3_. We created a flat surface on the tissue with a razor and applied cyanoacrylate-based superglue (Gorilla Glue, Cincinnati, OH, USA) to adhere the flat surface to a chilled vibratome stage (PELCO easiSlicer, Pelco, Redding, CA, USA). Additional sucrose cutting solution was used to submerge the brain tissue, which was then sliced into 300 μm sections that were transferred to a holding chamber with ice-cold, oxygenated artificial cerebrospinal fluid (aCSF) containing 125 mM NaCl, 3.5 mM KCl, 1.2 mM NaH_2_PO_4_ · H_2_O, 2.4 mM CaCl_2_ · 2H_2_O, 1.3 mM MgCl_2_ · 6H_2_O, 25 mM glucose, and 26 mM NaHCO_3_. The holding chamber with slices was then placed in a 35 °C water bath and attached to carbogen to be continuously oxygenated.

Individual slices were transferred from the holding chamber and placed on a perforated multielectrode array (MEA) (Multi Channel Systems MCS GmbH, Reutlingen, Germany), followed by suction of 0.1 mL/minute using a peristaltic pump (Multi Channel Systems). The MEA was continuously perfused with fresh aCSF buffer using a gravity perfusion system (VC-6-pinch, Warner Instruments, Hamden, CT, USA) at 35 °C with a heated perfusion cannula (Multi Channel Systems). Recordings were made using Multi Channel Experimenter (2.0, Multi Channel Systems) at 25 kHz. Treatments included a depolarizing version of the above aCSF with increased potassium chloride and no magnesium chloride (NaCl 125 mM, KCl 10 mM, NaH_2_PO_4_ · H_2_O 1.2 mM, CaCl_2_ · 2H_2_O 2.4 mM, glucose 25 mM, and NaHCO_3_ 26 mM), and regular aCSF with a bolus dose of tetrodotoxin (TTX, 10 nM). Each brain slice was habituated in the MEA and recorded for 30 min during normal aCSF perfusion (baseline), 30 min during perfusion with the depolarizing aCSF intended to elicit activity, and a final 30 min after a bolus dose of TTX at a final concentration of 10 nM and washout/perfusion with regular aCSF. The last 20 min of each treatment period were analyzed using Multi Channel Analyzer v2.0 (Multi Channel Systems) to allow the slices 10 min to stabilize and respond to treatments. Spikes were defined as drops in voltage at least 5 standard deviations below the average for each electrode.

### 2.10. Cloning and Plasmids

pBR-NL43-IRES-eGFP-nef+ (pBR43IeG) is a proviral vector containing an HIV molecular clone that expresses HIV-1 Nef and eGFP from a single bicistronic RNA and expresses a wildtype X4-tropic NL4-3 Env (obtained from the NIH AIDS Reagent Program #11349, Accession #M19921, contributed by Jan Münch, Michael Schindler, and Frank Kirchhoff [32,33]). p81A-4 is a full-length, HIV-1 infectious NL4-3 based molecular clone which expresses the V1-V3 regions of BaL Env (Obtained from the NIH AIDS Reagent Program #11440, contributed by Bruse Chesebro [34,35,36,37]). We generated the R5-tropic, BaL Env expressing reporter virus BR43IeG-BaL (hereafter referred to as HIV_BaL-GFP_) by cloning the *AgeI-BsaBI* fragment of p81A-4 containing the BaL Env sequence into the *AgeI-BsaBI* cut backbone of pBR43IeG (Figure S1). Plasmid DNA was large-scale purified using MidiPrep Kits (Qiagen, Hilden, Germany, #12143) and verified by sequencing (GeneWiz, South Plainfield, NJ, USA).

### 2.11. Preparation of Virus Stocks

HEK293T cells were transfected with HIV-1 proviral DNA using Lipofectamine 3000 (Invitrogen #L3000001) according to manufacturer’s recommendations. Virus-containing supernatants were collected at 48 and 72 h post-transfection, filtered through a 0.45 μm filter, and concentrated 10× by volume using Retro-Concentin reagent (System Biosciences, Palo Alto, CA, USA, #RV100A-1) according to manufacturer’s recommendations. Concentrated stocks were aliquoted and frozen at −80 °C. Virus stocks were quantified by measuring p24 gag protein by p24 alphaLISA (Revvity #AL291C) and were titered by infecting MDMs from healthy donors at two-fold serial dilutions and determining percent GFP+ cells by flow cytometry.

### 2.12. Infection of Producer Cells and Inoculation of Tissue Slices

At 48–72 h post-activation and differentiation, approximately 1 × 10^6^ CD4+ T-cells or MDMs, respectively, were infected with 0.1 μg or 7.2 μg p24 HIV_BaL-GFP_ virus stock, with the higher concentration being equivalent to the volume of virus that infected 10–25% of MDMs. At 48 h post-infection, uninfected and infected CD4+ T-cells were collected, washed, and counted, and we reserved approximately 0.1 × 10^6^ cells for flow cytometry. Uninfected and infected MDMs were washed and removed from the plate by incubation with cell dissociation buffer [PBS without Ca^2+^/Mg^2+^ + 2 mM EDTA (ethylenediaminetetraacetic acid, Fisher Scientific # BP2482100)] at 37 °C for 30 min, allowing us to count and reserve approximately 0.1 × 10^6^ cells for flow cytometry. T-cells and MDMs were resuspended in human slice culture media such that slices were inoculated with 10 μL of producer cells each, overlaid on top of the slice. Slices were distributed in wells as follows: 4× slices per well, per condition to determine GFP+ cells by flow cytometry (1× slice was processed per timepoint); 1× slice per well to quantify p24 and extract RNA.

Where indicated, slices were cultured in the presence of antiretrovirals (ARVs) added to the culture media at the time of cell-associated virus inoculation. We used combination ARVs that comprise the first-line therapy Biktarvy [38]: 33 nM bictegravir (BIC) (MedChemExpress, Monmouth Junction, NJ, USA, #HY-17605), 350 nM emtricitabine (FTC) (NIH AIDS Reagent Program #10071), and 5.4μM tenofovir alafenamide (TAF) (MedChemExpress #HY-15232B). These concentrations are all 4xEC_95_, which effectively inhibits viral replication [39,40]. ARVs were replenished in the slice culture media every two to three days, corresponding to collection days.

### 2.13. Quantification of Spreading Infection by p24 AlphaLISA

p24 gag was examined from slice culture media over time using p24 AlphaLISA according to manufacturer’s recommendations. Culture media was collected and replenished every two to three days post-infection for nine days total, and frozen until the end of the experiment. If interpolated p24 values fell below the standard curve, we assigned a value of “0.3”, corresponding to the lowest concentration of the standard curve. Inoculum-matched, uninfected background signal was subtracted from infected values per timepoint, per case. If this resulted in a value less than “0”, then we assigned “0”. Where indicated, values from infected slices cultured in the presence of ARVs were normalized to untreated slices. However, if both values were “0.3”, they were removed from the analysis. Values were excluded if they did not have a matched, uninfected control or if the viability performed in parallel indicated less than 80% viable cells.

### 2.14. RNA Extraction, cDNA Synthesis, and qPCR from Tissue Slices

Viral RNA from tissue slices was measured at day 9 post-infection. Slices were collected using a wet paintbrush and immediately lysed in Buffer RLT (Qiagen RNeasy Kit, Hilden, Germany, #74106) containing 10 μL/mL β-mercaptoethanol (Sigma #M3143) with vigorous vortexing. Lysates were either frozen at −80 °C or processed immediately according to manufacturer’s recommendations. Purified RNA was quantified by NanoDrop, normalized, and used to generate cDNA (Invitrogen SuperScript III First-Strand Synthesis System #18080051). cDNA was diluted 1:5 and 5 μL was used as template for qPCR in triplicate with SYBR Green Master Mix (ThermoFisher Scientific #4385612) and the following primers:
HIV-1 gag (0.8μM final): 5′ GGTGCGAGAGCGTCAGTATTAAG 3′
5′ AGCTCCCTGCTTGCCCATA 3′GAPDH (0.4μM final): 5′ GCTCACTGGCATGGCCTTCCGTGT 3′
5′ TGGAGGAGTGGGTGTCGCTGTTGA 3′

Samples were run on QuantStudio 7 real-time qPCR (Applied Biosystems, Waltham, MA, USA). If cycle numbers returned as “Undetermined”, we assigned a value of “40”, corresponding to the highest cycle number. ΔCt values were calculated from averages of technical triplicates of Ct values (gag-GAPDH). ΔΔCt values were calculated as 2^−(ΔCt infected − ΔCt uninfected)^. Where indicated, ΔCt values from infected slices cultured in the presence of ARVs were normalized to untreated slices. Values were excluded if the infected values were less than the uninfected control or if the viability performed in parallel indicated less than 80% viable cells.

### 2.15. Flow Cytometry

All cells were stained for viability using the live/dead stain, Zombie Yellow (Biolegend, San Diego, CA, USA, #423103), according to manufacturer’s recommendations, unless otherwise indicated (i.e., unstained). Briefly, cells were incubated with Zombie Yellow (1:1000) in PBS without Ca^2+^/Mg^2+^ for 10 min in the dark at 4 °C. The stain was diluted with PBS without Ca^2+^/Mg^2+^, cells spun at 1200 rpm for 5 min at 4 °C, and either fixed in 1% formaldehyde (Sigma 50-185-4042) or continued to be stained with extracellular and/or intracellular antibodies.

For PBMC characterization, cells were stained with antibodies against extracellular markers prepared in PBS without Ca^2+^/Mg^2+^: CD3 (AF700, BD Biosciences, Franklin Lakes, NJ, USA, Cat# 557917, RRID:AB_396938), CD4 (PerCP-Cy 5.5, BD Biosciences Cat# 552838, RRID:AB_394488), CD8 (BV786, BD Biosciences Cat# 563823, RRID:AB_2687487), CD14 (BV711, BD Biosciences Cat# 563372 (also 563373), RRID:AB_2744290), CD19 (AF647, Novus Biologicals, Minneapolis, MN, USA, #NBP2-61908AF647), and CD56 (PE-CF594, BD Biosciences Cat# 562328, RRID:AB_11153852) for 20 min in the dark at 4 °C. The antibodies and cells were diluted with PBS without Ca^2+^/Mg^2+^, spun at 1200 rpm for 5 min at 4 °C, and fixed in 1% formaldehyde. Data are calculated as the % of Singlets and Live cells.

For isolate purity, CD4+ T-cells and CD4-depleted PBMCs were stained with Zombie Yellow and antibodies against extracellular markers CD3 (AF700, BD #557917) and CD4 (PerCP-Cy 5.5, BD #552838) as above. Cells were fixed in 1% formaldehyde. Data were calculated as the % of Singlets, Live, CD3+, CD4+ cells.

For determination of infected producer cells, CD4+ T-cells and/or MDMs were stained with Zombie Yellow as above and fixed in 1% formaldehyde. Data are calculated as the % of Singlets, Live, GFP+ cells gated based on uninfected controls. Signals from uninfected samples were subtracted from infected samples.

For viability determination of brain tissue at indicated timepoints, dissociates were stained with Zombie Yellow and antibodies against extracellular markers CD45 (APC-H7, BD Biosciences Cat# 560274, RRID:AB_1645480), EAAT1/GLAST1 (AF405, Novus Biologicals #NB100-1869AF405), CD11b (BV711, BD Biosciences Cat# 568229, RRID:AB_2916857), TMEM119 (AF647, Novus Biologicals #FAB10313R-100ug), CD3 (AF700, BD Biosciences Cat# 557917, RRID:AB_396938), CD19 (AF700, BD Biosciences Cat# 561031, RRID:AB_10562552), and MBP (AF700, Novus Biologicals #NBP2-22121AF700) as above. Cells were then fixed and permeabilized using the Cytofix/Cytoperm kit (BD #554714) according to manufacturer’s recommendations. Briefly, cells were incubated in Fix/Perm buffer for 20 min in the dark at 4 °C. Cells were then washed 3x with 1× Perm/Wash buffer and were stained with antibodies against intracellular antigens prepared in 1× Perm/Wash buffer: histone H3 (FITC, Novus Biologicals #NB500-171F) or Ki67 (FITC, BD Biosciences Cat# 556026, RRID:AB_396302), NeuN (PE, Novus Cat# NBP1-92693PE, RRID:AB_11036146), GFAP (PerCP, Novus Biologicals #NBP2-34413PCP), Eno2 (PE-ATTO594, Novus Biologicals #NBP2-54452PEATT594), and β-III-tubulin (PE/Cy7, BioLegend Cat# 801217 (also 801218), RRID:AB_2876747) for 20 min in the dark at 4 °C. Cells were washed twice in 1× Perm/Wash buffer and fixed in 1% formaldehyde.

To measure infection of brain tissue at indicated days post-inoculation, dissociates were stained with Zombie Yellow and antibodies against extracellular and intracellular markers as for the viability determination with the following exceptions: GFP+ cells were measured instead of histone H3 or Ki67 and cells were stained for HIV-1 gag (PE, HIV Reagent Program #13449) instead of NeuN.

Cells were collected and characterized by the Cytek FACSort DxP12 flow cytometer and data were analyzed using FlowJo v10.8.1 software.

### 2.16. Gating Strategy and Population Calculations

Populations were gated based on unstained controls collected in parallel. The total live population is defined as Zombie Yellow negative, Singlets. The “dump” or “exclusion” channel eliminates CD3+ T-cells, CD19+ B-cells, and MBP+ Oligodendrocytes from our analysis. Astrocytes are defined as the population of cells that are GFAP+/CD11b-/Eno2−/NeuN− and CD3−/CD19−/MBP−. Myeloid cells are defined as the population of cells that are GFAP−/CD11b+/Eno2−/NeuN− and CD3−/CD19−/MBP−. Neurons are defined as the population of cells that are NeuN+/Eno2+/GFAP−/CD11b−/TMEM119−/EAAT1/GLAST1− and CD3−/CD19−/MBP−. The population of cells that are not astrocytes, myeloid cells, or neurons were defined using Boolean “not gates” for each of the cell-type specific populations as well as all other undefined populations (GFAP+/CD11b+, GFAP+/Eno2+, GFAP+/NeuN+, GFAP+/Eno2+/NeuN+, CD11b+/Eno2+, CD11b+/NeuN+, CD11b+/Eno2+/NeuN+, GFAP−/CD11b−/Eno2−/NeuN−/TMEM119+, GFAP−/CD11b−/Eno2−/NeuN−/EAAT1/GLAST1+, and GFAP−/CD11b−/Eno2−/NeuN−/TMEM119+/GLAST1+).

The gating strategy to determine infectivity of subcellular populations from infected, dissociated brain tissue changes slightly to identify neurons as the population of cells that are Eno2+/TUBB3+/GFAP−/CD11b−/TMEM119−/EAAT1/GLAST1−. Populations were initially gated based on unstained controls collected in parallel per timepoint. GFP+ cells were then determined based on gates set on the inoculum-matched uninfected controls set to <0.1% positive. Normalized GFP+ cells per case per timepoint per inoculum are calculated as the number of events within the GFP+ subpopulation divided by the sum of GFP+ astrocytes, myeloid cells, and neurons × 100.

### 2.17. Experimental Design and Statistical Analysis

This study used brain tissues from human donors of both sexes, which were pooled together in final analyses. Donor tissues were excluded from downstream analyses if we measured less than 80% total viable cells in flow cytometry studies. Biological replicates are values from different donors, while technical replicates are values from slice cultures made from the same donor tissue. Reported N values represent biological replicates (displayed on graphs as unique symbols) except for multi-electrode array experiments that used individual slices as N values due to the lower number of donor tissues examined. Figure legends report the exact N and statistical analyses for each experiment. Data were compiled and analyzed using GraphPad PRISM 10.1.2. One case and one condition from the viral RNA measurement experiment were identified as outliers by the ROUT method and excluded from the final analysis. Experiments comparing means of two independent groups were analyzed using an unpaired two-tailed *t*-test, while experiments comparing means of three or more groups were analyzed using one-way ANOVA with Dunnett’s post hoc. Experiments with two independent variables were analyzed using two-way ANOVA with Tukey’s post hoc. Multi-electrode array studies were analyzed by repeated measures one-way ANOVA with Tukey’s post hoc.

## 3. Results

### 3.1. Screening of Tissue Donors and Exclusion Criteria

**Figure 1:**
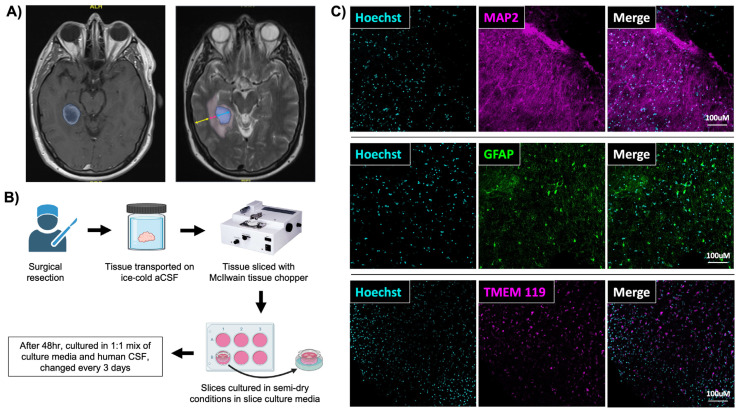
Donor screening and slice culture workflow. (**A**) Example MRI imaging of a suitable brain tissue donor with a deep brain pathology that required partial resection of cortical tissues to access. The left image shows a deep lesion in blue, while the right shows a similar section that highlights peri-lesional edema in pink. The right image also shows a multi-colored arrow demonstrating the optimal surgical path to the lesion that avoids eloquent brain. Our slice cultures only used the radiographically normal tissues marked by the yellow part of the incision path. (**B**) Flowchart of the slice culture protocol. Figure generated with BioRender. (**C**) Immunofluorescence staining of brain slices. Select brain slices were processed with a tissue clearing protocol and stained for either the neuronal marker microtubule-associated protein 2 (MAP2—magenta), the astrocyte marker glial fibrillary acidic protein (GFAP—green), or the microglia marker transmembrane protein 119 (TMEM 119—magenta). Slices were counterstained with Hoechst (cyan) to visualize cellular nuclei.

Demographic information of all patients who donated tissues for this study is presented in Table 1. Suitable patients for tissue donations generally presented with deeply situated pathologies that required corticectomy for optimal management. In these cases, we used brain tissues from the incision path of the surgery (further away from the pathology), which minimized confounding variables associated with pathology-adjacent tissues. All patients were radiographically screened to discern if tissues from the incision path were normal (Figure 1A), providing another level of control for tissue selection. Our studies only used tissues that met radiographic and clinical pathologic standards as being free of tumors, edema, reactive gliosis, and other pathology-associated phenotypes. All suitable patients gave their informed consent to donate tissues, including brain, blood, and cerebrospinal fluid, for use in this project.

### 3.2. Preparing Human Brain Organotypic Slice Cultures

Brain tissues used to create slice cultures were collected during normally scheduled brain surgeries from two different hospitals. Immediately after resection, we submerged brain tissues in cold artificial cerebrospinal fluid (aCSF) for transport to the laboratory. Brain samples were then cleaned, trimmed, and processed for same-day procedures (viability assays and/or MEA recordings) and long-term culture. We designed the brain slice culture protocol (Figure 1B) based on previously reported studies [41,42]. In brief, brain tissues were sectioned, sliced to 200 μm thickness with a McIlwain tissue chopper, separated, and plated at a density of 4–5 slices per well of a 6-well plate. Depending on the initial size and quality of the tissue, we routinely placed approximately 80–100 slices in culture per tissue donor. Brain slices were cultured in an air–liquid interface on semi-dry membrane inserts fed by human brain slice culture media in the lower chamber. The slices were robustly viable when cultured in human brain slice culture media for 48 h post-slicing followed by a 1:1 mixture of human brain slice culture media and human CSF (hCSF). Immunohistochemical staining of cleared brain slices showed extensive signals for neurons, astrocytes, and microglia, thereby providing a glimpse of the complex structural organization maintained in this system (Figure 1C). Moreover, tissue clearing revealed viable CNS cells throughout the tissue volume.

### 3.3. Adult Human Brain Tissue Slices Are Viable Ex Vivo for at Least Four Weeks in Culture

**Figure 2:**
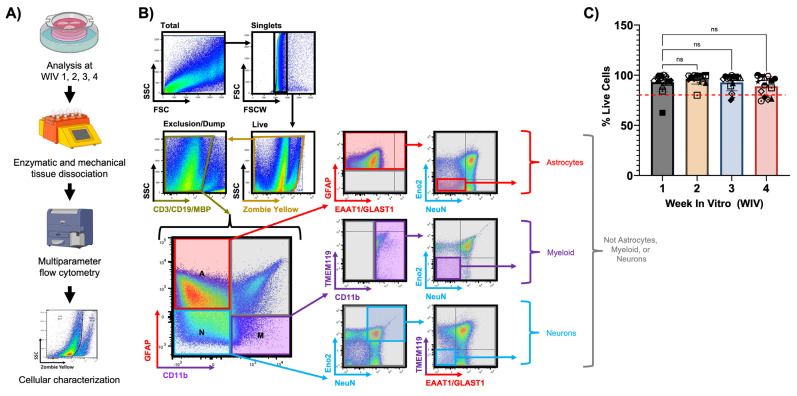
Cellular viability of slice cultures over time. (**A**) Flowchart describing the process of characterizing cellular viability and identifying CNS cell types over 4 weeks in vitro (WIV) via multiparameter flow cytometry. Figure generated with BioRender. (**B**) Hierarchical gating strategy for multiparameter flow cytometry. Example raw data shown from one case at WIV 2. (**C**) The overall cellular viability of slices shown as the percentage of live cells per week. The red dashed line indicates 80% viability. WIV 1–3, N = 15; WIV 4, N = 14) Independent cases are represented by unique symbols in the graph. Data plotted as mean ± SD and analyzed by one-way ANOVA with Dunnett’s post hoc.

We first determined the viability of human brain tissue slices over one month in culture using flow cytometry (Figure 2A). At weekly timepoints, we dissociated slice cultures into a single-cell suspension using enzymatic and mechanical disruption of the extracellular matrix, and then we removed any debris, myelin, and red blood cells from these samples. After gating for Singlets, the overall cell viability was measured using Zombie Yellow, an amine-reactive live/dead stain that labels cells with compromised plasma membranes (Figure 2B). The percentage of viable cells (Zombie Yellow-negative) remained very high throughout the four-week culture period, with only a modest, non-significant reduction in viable cells in the fourth week of culture (Figure 2C). Importantly, cell viability percentages were consistent within parallel slices from each case and across various sources of tissue, demonstrating that our culture system can support healthy brain slices for an extended period (Figure 2C). We also aimed to identify individual CNS cell types from the overall cell dissociate by staining and gating these preparations using at least two markers per CNS cell type, including GFAP and EAAT1 for astrocytes, CD11b and TMEM119 for myeloid cells, and NeuN, Eno2, and β-III-Tubulin for neurons. Our gating strategy (Figure 2B) first excluded aggregates, then included live cells (Zombie Yellow negative), and then excluded T cells (CD3 positive), B cells (CD19 positive), and oligodendrocytes (MBP positive), which left the samples with important CNS resident cell types. We then delineated astrocyte, myeloid, or neuron-containing populations by GFAP and/or CD11b, where we expect astrocytes in the GFAP-positive population (positive or negative for EAAT1), myeloid cells in the CD11b-positive population (positive or negative for TMEM119), and neurons in the GFAP-negative/CD11b-negative population. We further refined astrocyte and myeloid-containing populations as cells that were also negative for neuronal markers Eno2 and NeuN, and we also refined neuron-containing populations as cells that were negative for glial markers TMEM119 and EAAT1. This rigorous gating strategy provides high confidence that we can stringently identify astrocytes, neurons, and myeloid cells from the overall cell dissociate, but it cannot consistently distinguish microglia from macrophages, despite well characterized differences in CD11b and CD45 expression levels between the two cell types (Microglia: CD11b+/CD45^med^; Microglia: CD11b+/CD45^high^, [43,44]). This stringent protocol also produces a population of cells that fail to cleanly segregate into the three cellular populations, which may be due to loss of CNS cells membrane processes during tissue dissociation. Overall, our culture conditions preserve the major CNS cell types and promote high cellular viability for several weeks, which represents an ideal timeframe for downstream experiments.

### 3.4. Slice Cultures Maintain High Dendritic Spine Density with Mature Spine Morphologies

**Figure 3:**
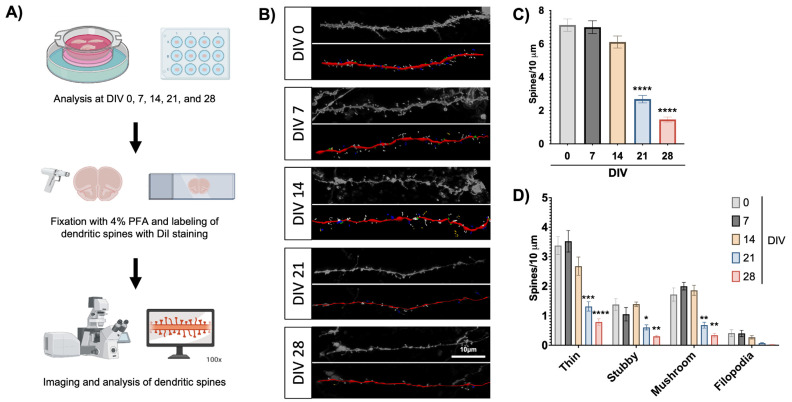
Dendritic spine density and morphology in slice culture. (**A**) Flowchart of dendritic spine labeling and analysis. Figure generated with BioRender. (**B**) Representative images of human dendrites stained with DiI (top, grayscale) and analyzed with Neurolucida 360 software (bottom, pseudo-color) from each experimental timepoint. Scale bar = 10 μm. Analyzed images highlight dendrites (red), filopodia (yellow), thin spines (white), mushroom spines (blue), and stubby spines (green). (**C**) Overall dendritic spine density over the culture lifetime. Each experimental timepoint examined at least 3 dendrites per slice from 3–4 slices. Data averaged from 3 cases and analyzed by one-way ANOVA with Dunnett’s post hoc, **** *p* < 0.0001. (**D**) Dendritic spine density from panel (**C**) broken down by spine morphology. Data analyzed by two-way ANOVA with Dunnett’s post hoc **** *p* < 0.0001, *** *p* < 0.001, ** *p* < 0.01, * *p* < 0.05.

Next, we examined slice cultures for dendritic spine density and morphology over time as an additional indicator of neuronal health over the culture lifespan (Figure 3A). Dendritic spines are post-synaptic sites that facilitate excitatory neurotransmission, mediate learning and memory processing, and are often lost in neurologic disorders [45], which makes them attractive targets in preclinical research. We examined dendritic spines in brain slices over four weeks in culture, as our flow cytometry studies suggest slice cultures remain viable through this period. At each timepoint, we stained brain slices with DiI, a fluorescent lipophilic dye that labels neuronal cell membranes and associated dendritic spines, allowing us to visualize these structures via confocal microscopy. Then, we analyzed the micrographs with Neurolucida360 software to characterize spines based on their morphology and quantify the density of spines along the dendrites, as shown in representative images of confocal and analyzed images (Figure 3B). The overall dendritic spine density in cultured brain slices remained quite robust for the first two weeks in culture but dropped at the end of the third week (Figure 3C), suggesting that spine changes represent an early sign of neuronal distress in our culture system. This is a highly valuable observation for the use of this culture system as a neuroHIV model since spine deficits are an important component of HAND [23,46,47]. Individual spine types followed the same trend, as the mature spines (thin and mushroom) remained robustly expressed for the first two weeks in culture and then dropped significantly at the three- and four-week timepoints (Figure 3D). This was also the case for immature spine types (stubby and filopodia), although only stubby spines showed a significant decrease at later timepoints.

These results align with our flow cytometry data that showed a general stability of the culture over the four weeks in culture (Figure 2). Taken together, these results further suggest that the first two weeks in culture are ideal for infection experiments.

### 3.5. Multi-Electrode Array Recordings of Acute Brain Slices

**Figure 4:**
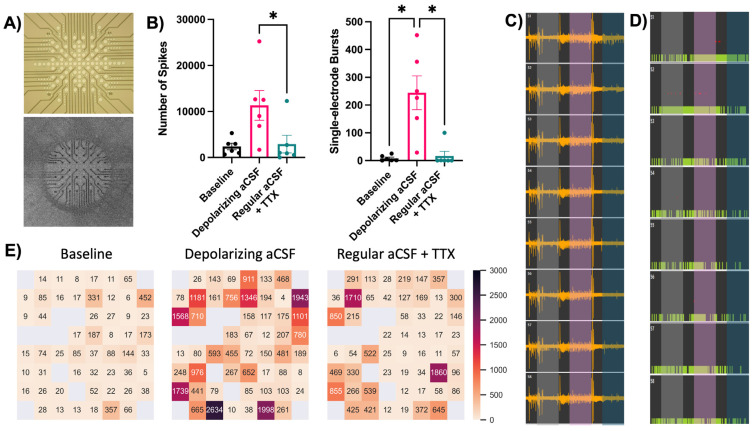
Acute slice activity recordings with multi-electrode arrays. (**A**) Schematic of multi-electrode array grid (top image), which contacts an acute brain slice (bottom image) to record local field potentials over time. (**B**) Neuronal activity quantifications. Tissues from three cases were recorded and analyzed for overall number of spikes (left) and the total number of single-electrode bursts (right) over the same baseline and treatment periods. N = 6 slices (2 slices per donor), data analyzed by one-way repeated measures ANOVA with Tukey’s post hoc, * *p* < 0.05. (**C**) Example voltage traces from one row of electrodes over an entire 90 min recording session. Colored overlays indicate 20 min recording periods analyzed at baseline (gray) or after treatments (depolarizing aCSF: pink, TTX: teal). (**D**) The same example voltage traces analyzed for voltage spikes (green bars) and single-electrode bursts (red dots) using Multi Channel Analyzer software v.2.18.0.21200. (**E**) Heatmaps from one recording session showing the number of spikes recorded at each electrode during the three analysis periods. Gray squares indicate electrodes that failed to record physiological values and were thus not representative of tissue activity.

The mature phenotypes and density of dendritic spines suggest that neurons in the brain slices remain active and functional over time. To gather another indication of neuronal health from the donated tissue, we also measured their activity with multi-electrode array (MEA) culture dishes. Our MEA dishes contain a 60-electrode grid (Figure 4A) that can record local field potentials of slice cultures, and this data can be further analyzed to identify and quantify spiking activity over time and during treatments. This experiment tested each slice’s baseline activity and response to depolarizing and suppressive treatments over a single 90 min recording period. The recording was organized in 30 min segments, where each segment featured an initial treatment (or aCSF perfusion for baseline) followed by a 10 min period to stabilize slice activity and a final 20 min period that was analyzed for spiking and bursting activity. This experiment examined tissues from three donors, and individual slices were excluded from final analyses if they failed to predictably respond to a depolarizing aCSF (with 10 mM K^+^, no Mg) or the suppressive treatment tetrodotoxin (TTX). Our recordings of baseline activity successfully detected individual spikes, and after slices were perfused during the second segment with depolarizing aCSF, treatment with TTX significantly decreased spiking activity in the third recording segment. We also found similarly significant results when the same recordings were analyzed for single-electrode bursts, which are consecutive spikes over a short period at a single electrode (Figure 4B). Treatment effects were also visible in raw voltage traces of the entire 90 min recording session of a single tissue, which are overlaid with colors that mark the 20 min analysis periods at baseline (gray) and after depolarizing aCSF (pink) and TTX (teal) treatments (Figure 4C). An analyzed version of these voltage traces shows expected trends for individual spikes (green) and single-electrode bursts (red dots), with the same color overlay for treatments (Figure 4D). We also used data from a single recording session to plot the number of spikes from each electrode in heatmaps, which demonstrate how spiking activity can vary across the electrode array (Figure 4E). The three heatmaps correspond with our three treatment conditions described above and clearly show the expected neuronal activity trends after each treatment. Variability between electrodes was similar to another study that used larger multi-electrode arrays to measure extracellular potentials of human tissues [42]. Overall, MEA recordings can detect local field potentials and bursting activity across multiple donor tissues, suggesting a promising approach to measure neuronal network activity in brain slice cultures.

### 3.6. Cell-Associated HIV-1 Infection of Ex Vivo Human Brain Slice Cultures

**Figure 5:**
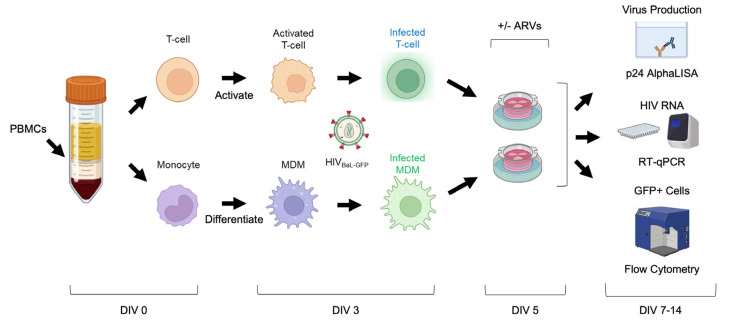
Schematic of slice culture infection protocol. Whole blood collected before brain tissue resection (DIV 0) was used to purify patient-matched CD4+ T-cells and monocytes. T-cells were then activated with PHA/IL-2 and monocytes were differentiated into MDMs with human serum and MCSF. At DIV 3, cells were washed, counted, and infected with HIV_BaL-GFP._ Then, at DIV 5, infected T-cells and MDMs were washed, counted, and used to inoculate their patient-matched slice cultures in the presence or absence of ARVs. From DIV 7–14, we tracked the slice culture media for viral p24 alphaLISA, and examined slices for viral RNA using qPCR, and GFP+-infected cell types using flow cytometry. Figure generated with BioRender.

We next designed experiments to infect brain slices with HIV-1 and track the infection within the first two weeks of culture (Figure 5). We utilized a GFP-expressing HIV-1 reporter virus HIV_BaL-GFP_, which was produced from the pBR43IeG-BaL molecular clone and based on the pBR43IeG backbone, which co-expresses eGFP from an IRES inserted into the Nef open reading frame [32,33] (Figure S1). We replaced the X4-tropic NL4-3 Env with an R5-tropic BaL Env to recapitulate physiologic infection of the CNS [34,35,36,37] (Figure S1). While preparing a new brain slice culture, we also isolated peripheral blood mononuclear cells (PBMCs) from donor-matched whole blood, purified CD4+ T-cells using positive selection beads, and collected monocytes via the unbound flow-through. We then activated the CD4 T-cell isolates with PHA-P and differentiated the monocyte isolates into macrophages (MDMs) via plastic adherence and MCSF. On average, CD4+ T-cell isolates were 85.7% pure, while 14.4% of CD4+ T-cells remained in the CD4-depleted PBMC population (Figure S2A). On these cells’ third day in culture, we inoculated both cell types with HIV_BaL-GFP_ (0.1 μg or 7.2 μg p24 per 1 × 10^6^ cells). HIV_BaL-GFP_ typically infected 1.03% of T-cells and 2.95% of MDMs after 48 h, as measured by live/GFP+ cells via flow cytometry (Figure S2B). On these cells’ fifth day in culture, we collected them in suspension and directly inoculated donor-matched brain slices in culture with T-cells (on average 3.25 × 10^5^ cells/slice) or MDMs (on average 1.38 × 10^5^ cells/slice) (Figure S2C). This allowed us to expose all slice cultures to a similar number of infected cells (on average 3 × 10^3^ infected T-cells or 1.7 × 10^3^ infected MDMs) (Figure S2D). We also treated select brain slices at the time of infection with the ARVs that comprise Biktarvy (bictegravir (BIC), 33 nM; emtricitabine (FTC), 350 nM; and tenofovir alafenamide (TAF), 5.4 μM) at a 4xEC_95_ concentration [48,49]. 4xEC_95_ concentrations are common in antiviral drug development and are expected to completely inhibit viral replication without significant toxicity [39,40]. Tissue or culture media was collected on days post-infection 2, 4, 7, and 9 (which correlates with slice culture DIV 7, 9, 12, and 14) to measure viral p24 in the culture media and GFP+-infected cells in the tissue. Viral RNA within the tissue was measured on day 9 post-infection (Figure 5).

### 3.7. Human Brain Slices Inoculated with Infected MDMs and T-Cells Can Support an HIV-1 Spreading Infection Ex Vivo

**Figure 6:**
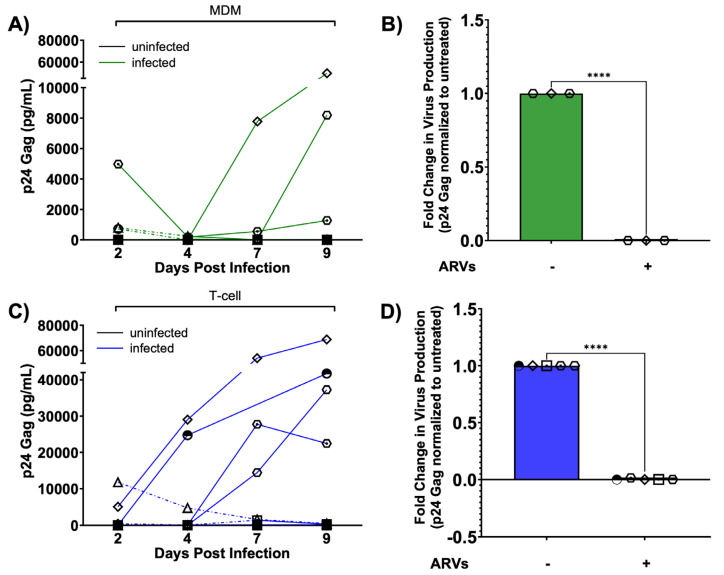
HIV replication kinetics in slice cultures. (**A**) MDM-based infections. Quantification of HIV p24 gag levels in culture media (pg/mL) with background subtracted (inoculum-specific uninfected) as a readout of viral replication kinetics over 9 days post-infection. Uninfected tissues are plotted as black lines (all at bottom of graph), while infected tissues are plotted as solid green lines indicating cases of spreading infection and dashed green lines indicating cases where infection failed to spread. (**B**) p24 gag levels from MDM-infected slices compared to parallel slices infected and treated with ARVs. Data shown normalized to untreated tissues from the same donor at day 9 post-infection. (**C**) T-cell-based infections. HIV p24 gag levels were quantified over time as in (**A**), except blue solid lines indicate spreading infection while blue dotted lines indicate cases where infection failed to spread. (**D**) Similar to panel B, p24 gag levels from T-cell-infected slices compared to parallel slices infected and treated with ARVs at day 9 post-infection. Data represent virus production from one slice per condition. Independent cases shown as unique symbols on the graphs; (**A**) Day 2, 4, 9, N = 9; Day 7, N = 8; (**B**): N = 3; (**C**): Day 2, 4, 9, N = 10; Day 7, N = 9; (**D**) N = 5. Data from panel (**B**,**D**) are plotted as mean ± SD and analyzed by two-tailed, unpaired *t*-test, **** *p* < 0.0001.

Following the experimental schematic presented in Figure 5, we determined if HIV-1-inoculated human brain slice cultures could propagate a spreading infection. We inoculated one slice per condition with infected T-cells or MDMs (or control, uninfected cells) in the presence or absence of ARVs, and then collected/changed culture media every 2–3 days for a total of nine days post-infection. We first measured the amount of p24 in the culture media as a proxy for virus production. In MDM-inoculated slices from three different cases (solid lines, out of nine total cases), we observed replication kinetics characteristic of a spreading infection, as p24 levels in the media mostly continued to increase up to day 9 post-infection (Figure 6A). In contrast, some MDM-inoculated slices did not support a detectable spreading infection (dashed lines, out of nine total cases) (Figure 6A). Similarly, T-cell-inoculated slices from five different cases (solid lines, out of ten total cases) showed increasing levels of p24 levels in the media, again consistent with a spreading infection (Figure 6C). Importantly, ARV-treated slice cultures produced significantly less p24 at day 9 post-infection regardless of the cell-type inoculum, further supporting that this system produces de novo infection of cells in the slice culture (Figure 6B,D). Our preliminary IHC staining also supports this point, as cells within slice cultures from one donor showed a strong p24 signal and associated viral GFP fluorescence at day 9 post-infection compared to uninfected slice cultures (Figure S3). Taken together, these results indicate that ex vivo human brain slices can be productively infected using donor-matched peripheral immune cells.

### 3.8. Infected Brain Slice Cultures Contain Viral RNA and Can Be Suppressed by ARVs

**Figure 7:**
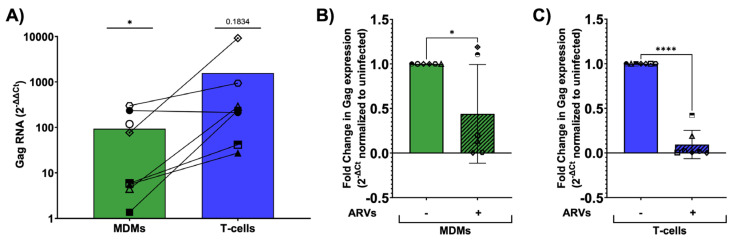
Viral RNA measurements from infected slice cultures. (**A**) ΔΔCt values were calculated comparing MDM-inoculated and T-cell-inoculated slices to uninfected controls. MDM, N = 8; T-cell, N = 7, data analyzed by two-tailed, unpaired *t*-test compared to uninfected, * *p* < 0.05. The fold change in gag expression in slices inoculated with MDMs (**B**) or T-cells (**C**) in the presence of ARVs (dashed bars) normalized to untreated (solid bars). Data plotted as mean ± SD of independent cases with individual data points per case indicated by unique symbols: (**B**) N = 6; (**C**) N = 7. Data analyzed by two-tailed, unpaired *t*-test, * *p* < 0.05, **** *p* < 0.0001.

We next used a more sensitive RT-qPCR method to detect viral RNA, aiming to verify infection in cultures with supernatant p24 and to detect possible low-level infection in the remaining cultures. We collected the control and inoculated slices at day 9 post-infection and isolated total RNA for downstream qPCR analysis. Viral RNA was quantified using HIV-1 gag specific primers and normalized to GAPDH. As expected, infected slices had increased levels of viral RNA regardless of the cell-type inoculation compared to uninfected control slices (Figure 7A). Furthermore, ARV-treated brain slices had significantly reduced expression of viral RNA compared to their untreated counterparts, regardless of the initial cellular inoculum in five of eight cases. ARVs suppressed viral RNA in four of six MDM-inoculated cases and all T-cell-inoculated slices (Figure 7B,C). Importantly, ARV treatment reduced viral RNA levels in cases that previously supported a spreading infection (Figure 7B, MDMs: diamond and hexagon; Figure 7C, T-cells: diamond, hexagon, and half-filled circle). In summary, viral RNA was present in cultures with and without detectable p24 in the media, and ARV treatment suppressed both viral RNA and p24 levels as expected.

### 3.9. HIV-1-Infected Slices Are Viable in Culture up to Nine Days Post-Infection

**Figure 8:**
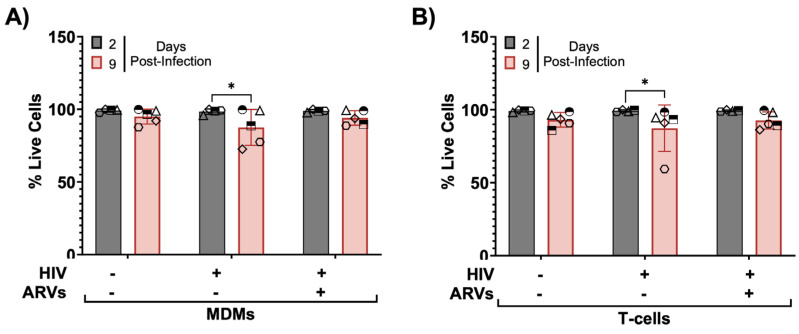
Tissue viability throughout infection and ARV treatment. Slices from (**A**) MDM-infected tissue or (**B**) T-cell-infected tissues analyzed for viability by flow cytometry. Gating was performed as indicated in Figure 2. Data per case represent dissociated cells from one slice per condition per timepoint. Data plotted as mean ± SD of independent cases (shown as unique symbols) and analyzed by two-way ANOVA with Tukey’s post hoc * *p* < 0.05; N = 5.

Next, we examined if HIV-1 infection affected the cellular viability within slice cultures using flow cytometry. We inoculated cultured brain slices with infected donor-matched cells as done before, dissociated tissues at days 2 and 9 post-infection, and determined their cellular viability using Zombie Yellow staining (Figure 8A,B). Both timepoints showed robust cellular viability with no significant differences between uninfected and infected slices, regardless of inoculum. However, the HIV-1-inoculated slices showed slightly less viability at day 9 post-infection compared to day 2 post-infection, which was not the case in ARV-treated slices (Figure 8A,B). Taken together, these data suggest that our slice culture system could indeed model how live human brain cells adapt to HIV infection and/or ARVs in a physiologically relevant tissue system.

### 3.10. HIV-1-Infected, GFP+ Astrocytes and Myeloid Cells Are Present in Human Brain Tissue Inoculated with Infected MDMs and T-Cells

**Figure 9:**
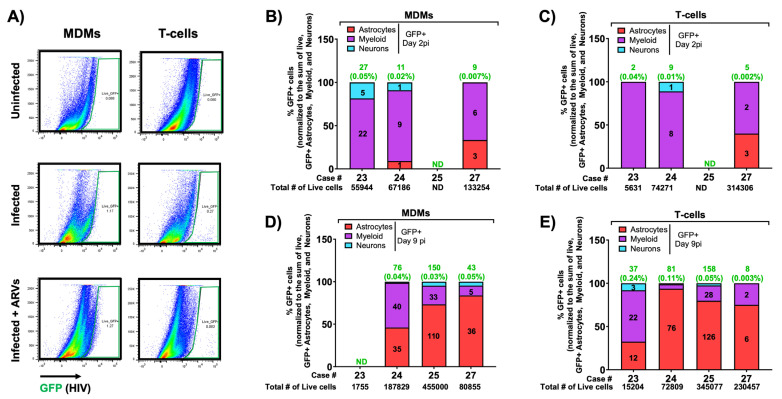
Identification of GFP+-infected cells from inoculated slices. Ex vivo human brain slices were inoculated with HIV-1-infected MDMs (**A**,**B**,**D**) or T-cells (**A**,**C**,**E**), collected at days 2 and 9 post-infection, and analyzed for infected subcellular populations via flow cytometry. (**A**) Example dot plots from live GFP+ cells from one case at day 2 post-infection, indicating GFP+ gating from uninfected slices or those inoculated with infected MDMs or T-cells (green) with or without ARV treatment. Gating was performed as indicated and subcellular populations defined as in Figure 2B (astrocytes—red; myeloid—purple; neurons—blue). The %GFP+ cells of the indicated populations were determined by gating on the matched uninfected inoculum such that the GFP+ percentage of the control was ≤0.1%. GFP+ cell counts from each subcellular population per case per timepoint per inoculum were normalized to the sum of GFP+ astrocytes, myeloid cells, and neurons. “ND” is not detected and is indicated when values are less than 0.001%. Green numbers above each bar indicate the raw counts of GFP+ cells and the associated percent of the total number of live cells (indicated in black below the x-axis). Black numbers within each bar indicate the raw counts of GFP+ cells within each subpopulation. (**B**) MDM-inoculated slices at day 2 post-infection, N = 3; (**C**) T-cell-inoculated slices at day 2 post-infection, N = 3; (**D**) MDM-inoculated slices at day 9 post-infection, N = 3; (**E**) T-cell-inoculated slices at day 9 post-infection, N = 4. Data per case represent dissociated cells from one slice per condition per time point.

Since the HIV_BaL-GFP_ virus expresses GFP in infected cells, we next determined which CNS cell types were infected using flow cytometry with an infectivity-specific gating strategy. This strategy differs from our viability strategy slightly by detecting GFP and HIV p24 gag instead of Ki67 or histone H3 and NeuN, respectively. However, we still discriminate neuron-containing populations via Eno2/TUBB3 double-positive cells. Total tissue dissociates were analyzed by flow cytometry as in Figure 2B to discriminate astrocyte, myeloid, or neuronal populations, but then astrocyte and myeloid-containing populations were further refined as those that lacked neuronal markers Eno2 and TUBB3. We first established all gates on unstained controls, then analyzed GFP+ cells in the astrocyte, myeloid, and neuron-containing populations based on inoculum-matched uninfected controls (set to <0.1%) (Figure 9A). As we initially excluded CD3+ cells, this effectively eliminated any GFP signal from residual producer T-cells, therefore adding confidence that the GFP signal is from de novo infected cells of the CNS. GFP+ cells from MDM and T-cell-inoculated slices were first quantified at day 2 post-infection, a timepoint that showed very few GFP+ cells overall (0.02–0.07% of cells in MDM-inoculated slices, 0.002–0.04% of cells in T-cell-inoculated slices) (Figure 9B,C). This small number of GFP+ cells mostly fell within the myeloid-containing populations (Figure 9B,C). At day 9 post-infection, brain slices had significantly more GFP+ cells in their dissociates (average of 0.04% of cells from MDM-inoculated slices, 0.003–0.24% of cells from T-cell-inoculated slices), further suggesting viral spread over time (Figure 9D,E). At the same timepoint, we also measured GFP+ cells from the myeloid cell population and an increased presence of infected astrocytes, with a minor signal from the neuron-containing population as well, which likely indicates the background signal of this assay. Overall, our brain slice culture system supported a spreading infection that targeted mostly expected cell types within the CNS while remaining highly viable, suggesting that this system could serve as an outstanding human-based model for HIV-1 neuropathogenesis.

## 4. Discussion

This study is the first to report a tractable model of HIV-1 brain infection based entirely on healthy, intact adult human brain tissues in organotypic slice culture. This new model is a significant advance in the field, as it has the potential to overcome long-standing issues with animal models that lack the structure and complexity of the human brain and to better identify therapeutic approaches for HAND in the preclinical stage. The model is made possible by tissue donors undergoing brain surgeries, which provide us radiographically normal, healthy tissues that are distant from the targeted surgical lesion. Importantly, resected brain tissues are immediately preserved in cold aCSF and transferred to the laboratory, thus maximally preserving tissue structure and health. The brain slice cultures are viable for several weeks, they contain all the major CNS cell types and preserve their biological complexity, and they demonstrate both structural and functional aspects of healthy neuronal networks. We also provide one of the first reports of a highly stringent human CNS-specific flow cytometry panel to characterize cells from human brain tissue. Most importantly, we can infect brain slice cultures with a GFP-expressing HIV-1 using the same tissue donor’s peripheral immune cells as trojan horses, thereby reflecting how HIV-1 likely infects the brain in vivo. This approach produces a spreading infection that can be measured by multiple techniques (including within individual cells), and the infection is fully suppressed by current first-line antiretroviral therapy. Overall, this adult, human brain-based model of neuroHIV is an important step forward that will facilitate future studies on mechanistic drivers of HAND/neuroHIV, drug target discovery, and preclinical treatment efficacy in human tissues.

The brain slice culture protocol we employed was informed by work from another group that optimized and characterized human brain slice cultures using similar surgical resections from epilepsy patients. This group substantially improved culture viability by adding hCSF to the culture media [50] and robustly characterized neuronal health and activity via electrophysiology and other approaches [41], lending confidence that optimized human brain slice cultures indeed preserve the complexity and function of human cortical tissues. We also included hCSF in our culture media and achieved high cellular viability and neuronal dendritic spine density over several weeks, further validating the human brain slice culture system. Notably, our study only used hCSF from a single donor, which eliminated this aspect as a source of variability. Our system could also detect local field potentials from these cultures using multi-electrode arrays, in line with another recent paper on such methods [42]. Together, these data suggest that human brain resections from the incision path of a surgery produce healthy, functional, long-term slice cultures regardless of the underlying patient pathology (epilepsy or a deep brain tumor).

Since our tissue donors present with pathologies that require different surgical paths and must meet selection criteria, one limitation of our system is that we receive heterogeneous cortical areas to use for slice cultures. However, as studies proceed and our sample size increases, these heterogeneous tissues may turn into an advantage as the outcomes from various brain regions could reveal important region-specific mechanistic differences that may also be relevant to HAND or other CNS pathologies. Future neuroHIV studies in this model should include cultures with detectable supernatant HIV p24 and tissue-associated viral RNA, as this strongly verifies the infection. In our study, three of the five slice cultures that produced detectable supernatant p24 were from the frontal cortex, suggesting a possible brain region susceptibility to infection ex vivo. However, cultures treated with antiretrovirals had suppressed viral RNA regardless of brain region or supernatant p24 levels, suggesting we achieved at least a low-level infection in all cases. These data are quite encouraging, as the GFP-encoding HIV-1 used to establish our model may not be as fit as other viral strains lacking the GFP sequence. Therefore, future studies with this model will most likely show infection from viral strains without the GFP sequence, including CNS-tropic strains and peripheral strains.

Additionally, pooled data from our slice culture system is well equipped to detect common physiological and disease mechanisms that are preserved throughout the brain, which could translate to more broadly relevant CNS drug targets. Importantly, our system only used healthy cortical tissue that was distant from deeper pathologies and radiographically normal, which limits confounds that may be present in pathology-adjacent tissues. Retrospective analyses of frozen tissue from these specimens showed negligible to no infiltration of inflammatory cells, further suggesting our cultures lack this major confounding variable.

Our work to validate and adapt adult human brain slice cultures to model HIV-1 infection is a critical contribution to preclinical HIV research. Though valuable, most preclinical studies of HIV-1 brain infection currently rely on either small animal models that only express a subset of viral proteins, humanized mice that do not fully reconstitute human CNS cells and architecture, or SIV-infected macaques, which do not fully reflect HIV-associated neurocognitive pathologies [51,52,53,54]. Some HIV-1 studies take advantage of brain organoids made of human CNS cells [55,56,57], but these systems fail to recapitulate the structure of the human brain and lack its cellular diversity [58]. Furthermore, they require addition of microglia from an external source or derive microglia from induced pluripotent stem cells [59], which is an important caveat since microglia remain as HIV reservoirs in people taking antiretroviral therapies [60]. Other groups have studied postmortem brain tissues from people with HIV, and although these retrospective studies provided important insights, they have so far mostly examined tissues from people with AIDS or HIV encephalitis that are less relevant to the modern era [61]. Modern neuroimaging approaches overcome some of these limitations [62], but they are not yet sensitive enough to measure subtle structural changes in neurons that likely contribute to impairment, for example, dendritic spine density [23]. To date, the field has lacked a physiological model of HIV-1 infection in live human CNS tissue, with the exception of one study that created a co-culture system using an HIV-infected cell line and human fetal brain slice cultures [63]. However, our system is much more relevant to the modern pathology, as we use adult human brain tissues, we infect the tissues with the same donor’s primary immune cells instead of a cell line, we detect HIV infection in the brain tissue itself, and we can suppress the infection using antiretrovirals. No other experimental systems in the field provide the same level of experimental opportunities while directly modeling the presumed route of brain infection and the overall complex structure of the human brain. We are continuing to improve the culture protocol to examine HIV infection longitudinally with new approaches to minimize cellular stress after brain resection and reproduce the brain’s oxygen and nutrient gradients in culture. These kinds of innovations will build on an already promising slice culture model of neuroHIV with strong potential to move the field forward.

A few other groups have published adult human brain slice culture models of viral infections, though they each have important caveats. For example, one group published a free-floating slice culture model to study Oropouche virus infection with human brain surgical resections [64], but their approach only maintained culture viability for a few days, therefore limiting the model’s utility. Another group recently published an approach to culture postmortem adult human cortical tissue slices as a model to study Tahyna virus infection [65]. Though this system allows investigators to choose the cortical region they wish to culture and study, the downside is the postmortem interval before collecting tissue ranged between 2–12 h. This long interval of little to no oxygenation likely changes the physiological nature of the brain [66], which may confound the results and reduce the benefits of culturing slices from similar cortical areas. Our slice culture system overcomes these downsides, as we immediately preserve live surgical resections in cold aCSF, we slice and culture all tissues within 2–3 h of resection, and the cultures remain highly viable for weeks using an optimized protocol. Therefore, our system is more likely to model the normal state of the brain and will allow longer-term investigations of viral infection and progression in healthy tissue, which will likely be important to accurately model disease mechanisms and discover relevant drug targets. In the future, we expect adult human brain slice cultures to be further adapted and developed to study a variety of pathologies and brain infections [67].

## 5. Conclusions

Our human brain slice model of HIV-1 infection is a significant step towards improving the experimental models available for preclinical neuroHIV studies as well as our understanding of the molecular and cellular mechanisms that drive disease in human tissues. The model also provides an unmatched approach for interventional studies in human tissues, which would typically only occur during clinical trials. Future work using this system is poised to overcome the long-standing limitations of animal models used in neuroHIV research and possibly discover a much-needed adjuvant strategy to treat modern forms of HAND that present despite viral suppression by antiretroviral therapies.

## Figures and Tables

**Table 1:** Tissue donor demographics.

**Age**	**N = 18 Cases**
Mean (SD)	57.6 (14.2)
Median	58
Range	33, 82
**Sex, n (%)**	
Female	11 (61.1)
Male	7 (38.9)
**Tissue collected, n (%)**	
Supratentorial neocortical parenchyma	18 (100)

## Data Availability

All data reported in this manuscript are available to qualified researchers upon reasonable request.

## Supplementary Materials

The following supporting information can be downloaded at: https://www.mdpi.com/article/10.3390/cells13131127/s1, Figure S1: Cloning and validation of the HIV-1 molecular clone pBR43IeG-BaL (BaL-GFP); Figure S2: Characteristics of cell isolation and infection; Figure S3: Immunohistochemical staining for HIV p24 in human brain slice cultures.
