# Supplementary material for: Adult Human Brain Tissue Cultures to Study NeuroHIV
Source: Cells. 2024 Jun 29;13(13):1127. doi: 10.3390/cells13131127 (PMC11240386; doi:10.3390/cells13131127)
Supplement: Supplementary file 1 [file cells-13-01127-s001.zip › cells-2990593-supplementary.pdf]

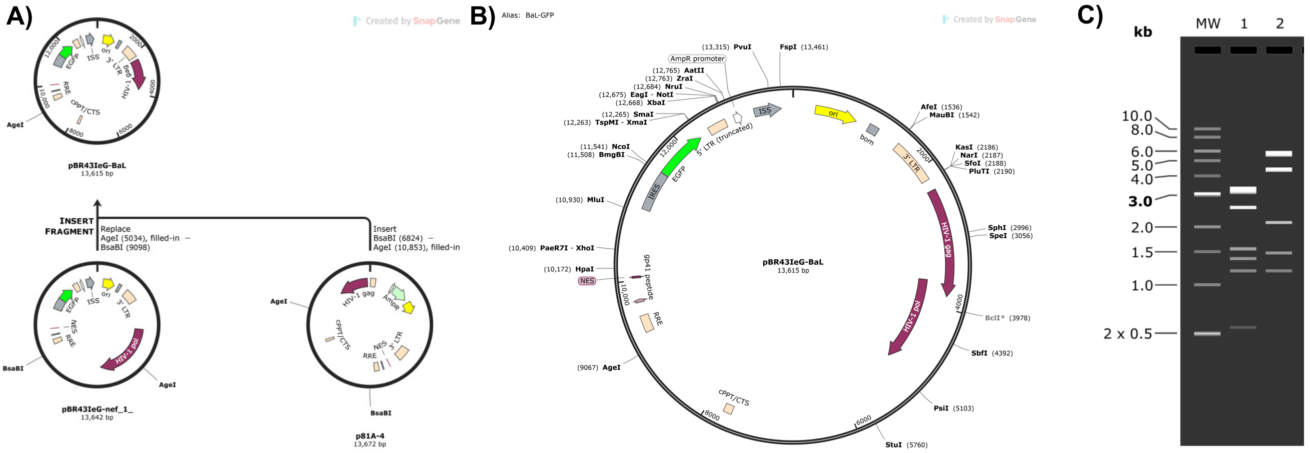

Supplemental Figure S1. Cloning and validation of the HIV-1 molecular clone pBR43IeG-BaL (BaL-GFP). **A)** The cloning strategy to generate the pBR43IeG-BaL HIV-1 molecular clone used in this study. The vector, pBR43IeG, is a pNL4-3-based, full-length HIV-1 molecular clone which expresses GFP from an IRES inserted downstream of the Nef ORF. The insert, p81A-4, is also a full-length HIV-1 molecular clone that expresses the V1-V3 loops of the BaL envelope. To generate the pBR43IeG plasmid, the AgeI-BsaBI fragment of pBR43IeG was replaced with the AgeI-BsaBI fragment of p81A-4. **B)** The resulting plasmid map of pBR43IeG is shown. **C)** Predicted HindIII diagnostic digests of pBR43IeG-BaL (lane 1) and pNL4-3 WT (lane 2) simulated relative to a 1kb Plus DNA ladder on a TAE gel. Figure created using SnapGene.

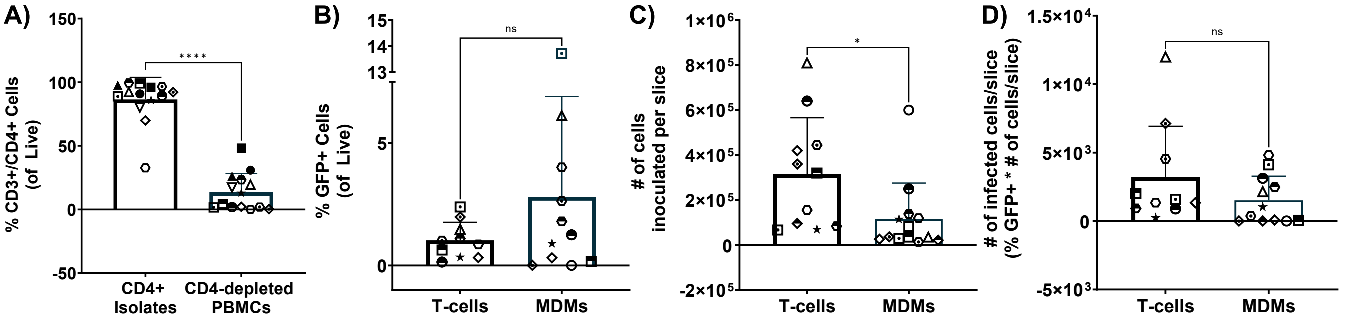

Supplemental Figure S2. Characteristics of cell isolation and infection. Ficoll density purification of PBMCs from whole blood collected on the day of tissue resection was used to isolate CD4+ T-cells using positive selection. CD4-depleted PBMCs containing monocytes were also reserved. **A)** Isolate purity was determined by live/dead, and antibody staining of ~1x105 cells from each population with Zombie Yellow, CD3+, and CD4+ antibodies. Data shown indicate the % live cells that are CD3+ and CD4+ in each population as determined by flow cytometry. Data shown are means ± SDs of independent cases (N=14). **B)** Infected CD4+ T-cells and MDMs were collected on DIV 5, Day 2 post-infection and ~1x105 cells were reserved to determine the percent infected producer cells by live/dead staining and GFP fluorescence as determined by flow cytometry. Data shown are means ± SDs of independent cases (T-cells, N=10; MDMs, N=11). **C)** On DIV 5, Day 2 post-infection, infected CD4+ T-cells or MDMs were used to inoculate slices. The number of CD4+ T-cells or MDMs added per slice are indicated. Data shown are means and SDs of independent cases (T-cells, N=10; MDMs, N=11). **D)** The number of infected cells added per slice were calculated by the percent GFP+ cells (B) multiplied by the # of cells added per slice (C) (T-cells, N=10 and MDMs, N=11). Individual data points per case indicated by unique symbols. Data analyzed by unpaired, two-tailed Student's t-test; **** p<0.0001.

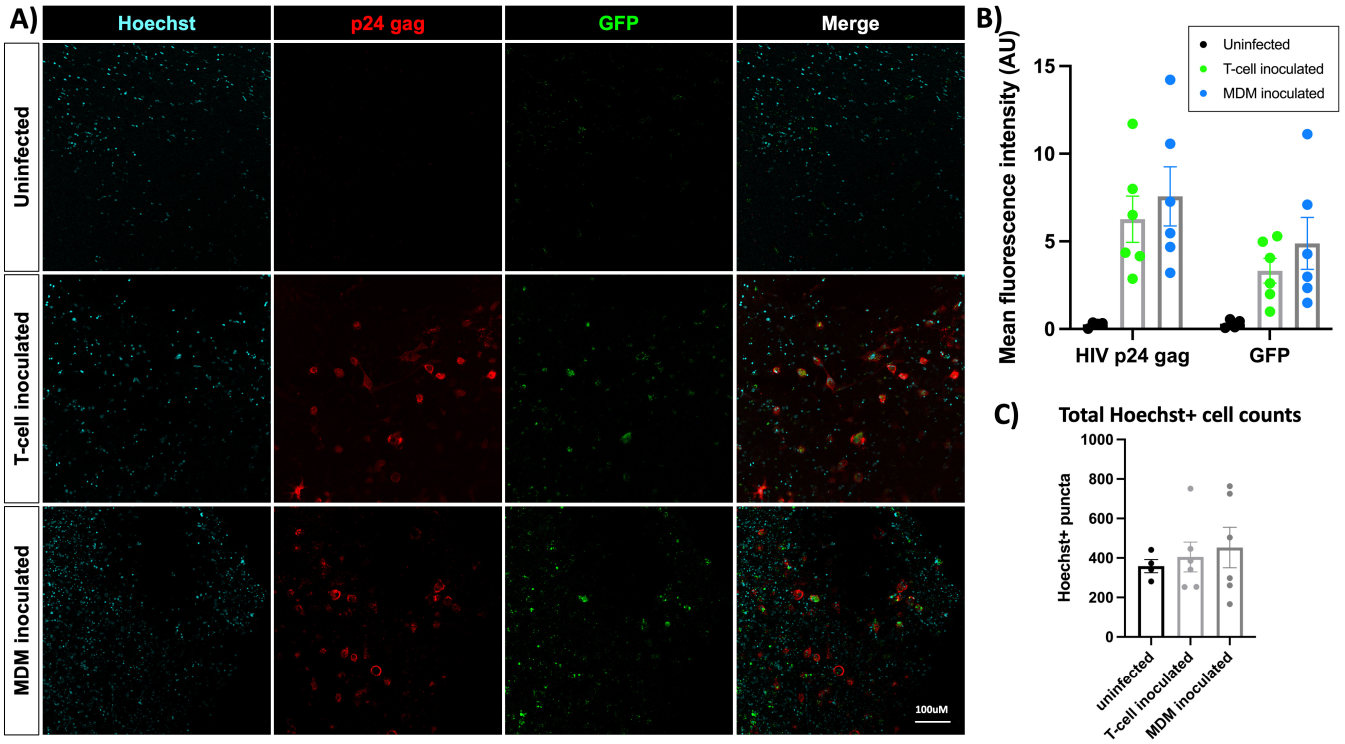

Supplemental Figure S3. Immunohistochemical staining for HIV p24 in human brain slice cultures. **(A)** Representative IHC images from day 9 post-infection slice cultures stained for HIV p24 gag and counterstained with Hoechst. The GFP signal was from direct laser excitation of GFP from $HIV_{BaL\text{-}GFP}$ infected cells. **(B)** Quantified mean fluorescence intensity of p24 gag and GFP from entire micrographs of infected and uninfected tissues. **(C)** Overall counts of Hoechst+ cells from the same micrographs analyzed in (B). All micrographs taken from random fields using a 20X objective and quantified using FIJI software (ImageJ). N=1 donor, dots indicate values from separate micrographs (n=4 for uninfected, n=6 for infected) for each treatment condition. Data shown as mean ± SEM.
